# Miniaturizing Controlled-Source EM Transmitters for Urban Underground Surveys: A Bipolar Square-Wave Inverter Approach with SiC-MOSFETs

**DOI:** 10.3390/s25134183

**Published:** 2025-07-04

**Authors:** Zhongping Wu, Kuiyuan Zhang, Rongbo Zhang, Zucan Lin, Meng Wang, Yongqing Wang, Qisheng Zhang

**Affiliations:** 1 School of Geophysics and Information Technology, China University of Geosciences (Beijing), Beijing 100083, China; wuzhonghping016@163.com (Z.W.); 2110220024@email.cugb.edu.cn (R.Z.); linzucan@hotmail.com (Z.L.); wangmeng@cugb.edu.cn (M.W.); wangyq@cugb.edu.cn (Y.W.); zqs@cugb.edu.cn (Q.Z.); 2 Key Laboratory of Intraplate Volcanoes and Earthquakes, China University of Geosciences (Beijing), Beijing 100083, China

**Keywords:** high-frequency electromagnetic transmitter, SiC-MOSFET, bipolar square-wave inversion, EMI testing

## Abstract

This paper presents a compact, high-efficiency electromagnetic transmitter for Controlled-source Audio-frequency Magnetotelluric (CSAMT) applications, operating in the 10–100 kHz range. A novel bipolar square-wave inverter topology is proposed, which directly modulates the transformer’s secondary-side AC output, eliminating conventional rectification and filtering stages. This design reduces system losses (simulated efficiency > 90%) and achieves an approximately 40% reduction in both volume and weight. The power stage uses a full-bridge bipolar inverter topology with SiC-MOSFETs, combined with a high-frequency transformer for voltage gain. Simulation, laboratory testing, and EMI evaluation confirm stable square-wave generation and full compliance with EN55032 Class A standards. Field validation with a CSAMT receiver demonstrates effective signal transmission and high-resolution subsurface imaging, thereby improving the efficiency and portability of urban geophysical exploration.

## 1. Introduction

As urbanization accelerates, the development of underground space has become a key strategy to alleviate land resource shortages and enhance urban resilience [1,2,3]. In shallow to intermediate-depth geophysical investigations (ranging from tens to hundreds of meters), the Controlled-source Audio-frequency Magnetotelluric (CSAMT) method transmits artificially generated electromagnetic waves at specific frequencies and analyzes the subsurface media’s response, thereby delineating geological structures [4,5,6]. Due to its high sensitivity to resistivity variations and high vertical resolution, CSAMT is widely used in applications such as active fault identification [7] and underground pipeline damage detection [8,9,10].

**Figure 1:**
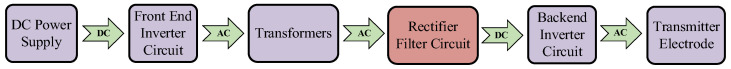
Conventional ultra-audio transmitter module diagram.

In such systems, the performance of the transmitter directly determines the detection accuracy. Conventional transmitters often adopt a topology that combines a boost converter with an inverter circuit, as illustrated in Figure 1. During the conversion of the input signal through multiple stages of voltage boosting and inversion, excessive waveform transformations result in substantial energy losses (typical efficiency is only 82–87% [11]). Moreover, the rectification and filtering stages require bulky capacitors and inductors that increase the transmitter’s volume and weight. The substantial weight of geophysical transmitters, exemplified by the GGT-30 unit (93 kg) manufactured by Zonge International (UK) and the TXU-30 unit (52.5 kg) produced by Phoenix Geophysics (Canada), significantly impedes field deployment efficiency during geophysical surveys.

In recent years, advancements in wide-bandgap semiconductors (such as SiC-MOSFETs) and high-frequency magnetic core materials have opened new avenues for transmitter miniaturization [12,13,14]. However, existing studies have mostly focused on optimizing individual components—such as implementing soft-switching techniques [15,16] or integrating magnetic components [17]—without addressing the systemic challenges of multi-stage power conversion and filtering.

In this work, A high-efficiency, miniaturized transmitter based on a bipolar square-wave inverter circuit was proposed. Its fundamental frequency range is 10 to 100 kHz. The key innovations are as follows:

(1) By directly inverting the AC voltage output from the transformer using a bipolar square wave inverter circuit, the rectification and filtering stages are eliminated, reducing energy loss and achieving a 70% reduction in system volume and weight compared to traditional high-frequency transmitters.
(2) The utilization of SiC-MOSFETs combined with transformers enables a reduction in the required number of winding turns or the magnetic core cross-sectional area for the transformer design under identical output power and other constant parameters as the operating frequency increases [18]. This facilitates a 40% reduction in weight and volume, consequently enhancing power density.
(3) A complementary timing control strategy with optimized dead time is introduced to ensure circuit stability under filterless operation. Simulation and experimental results demonstrate that the proposed system can reliably output square-wave signals in the 10–100 kHz range with efficiencies of up to 92%, providing a lightweight, reliable solution for urban subsurface exploration with millimeter-scale resolution.

## 2. Theoretical Basis for High-Frequency Transmitter Design

In Controlled-source Audio-frequency Magnetotelluric (CSAMT) survey systems, alternating current signals are injected into the ground to generate electromagnetic fields. Variations in apparent resistivity are then analyzed to infer subsurface structural features or detect anomalous bodies. According to the theory of skin effect, lower-frequency electromagnetic signals penetrate deeper geological layers, while higher-frequency signals are more effective for shallow subsurface imaging [19,20].

**Figure 2:**
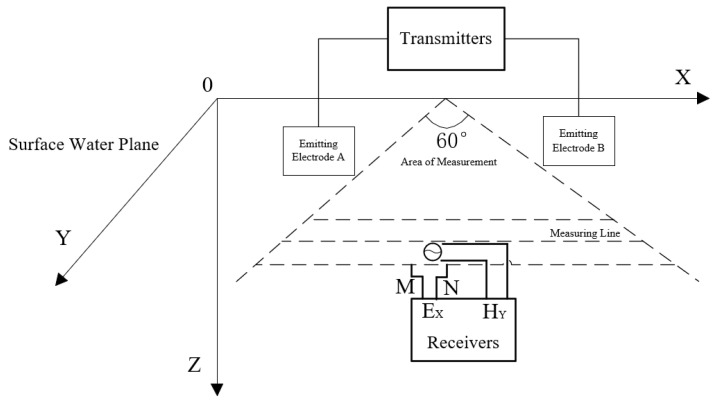
Ultra-audio Controlled-source Electromagnetic Detection schematic diagram.

As illustrated in Figure 2, a Cartesian coordinate system is established for the survey: the orthogonal X and Y axes lie on the horizontal surface, while the *Z*-axis points vertically downward [21,22]. The receiver measures the horizontal electric field component E and the vertical magnetic field component H. Based on Maxwell’s equations and electromagnetic wave propagation theory, the expressions for the horizontal electric field and the vertical magnetic field can be derived, as shown in Equations (1) and (2).

(1) $$E_{x}=\frac{Il\rho }{2\pi r^{3}}\left(3\cos\varphi -2\right)$$

(2) $$H_{y}=\frac{Il}{2\pi r^{3}\sqrt{-i\omega \rho \sigma }}\left(3\cos\varphi -2\right)$$

In the above equations, ρ represents the resistivity of the subsurface medium, I denotes the transmitted current amplitude, l is the electrode spacing, and r is the distance between the transmitter and receiver. Based on Equations (1) and (2), the single-component apparent resistivities $\rho_{B_{x}}$ and $\rho_{H_{y}}$ can be calculated using the following expressions, as shown in Equations (3) and (4).

(3) $$\rho_{B_{x}}=\frac{4\pi r^{3}}{Il}\left|\frac{E_{x}}{3\cos2\varphi -1}\right|$$

(4) $$\rho_{H_{y}}=\frac{16\pi^{2}\mu \omega r^{6}}{I^{2}l}\left|\frac{H_{y}}{3\cos2\varphi -1}\right|$$

By computing the ratio of the horizontal electric field to the vertical magnetic field, the Cagniard apparent resistivity $\rho_{c}$ of the subsurface material can be derived.

(5) $$\rho_{c}=\frac{1}{\omega u_{0}}{\left|\frac{E_{x}}{H_{y}}\right|}^{2}$$

where $\omega =2\pi f_{0}$, $f_{0}$ is the frequency of the electromagnetic field, and $u_{0}$ is the magnetic permeability of free space, with $u_{0}=4\pi \times 10^{-7}\frac{H}{m}$.

In practical applications, to simplify calculations, the exploration depth can be estimated using an empirical formula based on the apparent resistivity and signal frequency:

(6) $$D\left(f\right)=356\sqrt{\frac{\rho_{c}}{f}}$$

where D(f) represents the Bostick investigation depth, and f is the transmission frequency. Based on this, the formula for calculating vertical resolution can be derived.

(7) $$\Delta D=D\left(f_{1}\right)-D\left(f_{2}\right)=356\sqrt{\rho_{c}}\left(\frac{1}{\sqrt{f_{1}}}-\frac{1}{\sqrt{f_{2}}}\right)$$

where $f_{1}$ and $f_{2}$ are any two adjacent frequencies.

**Figure 3:**
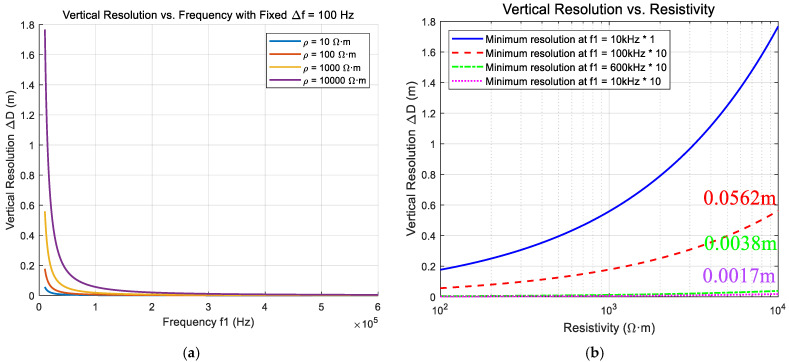
Relationship between frequency and resistivity. (**a**) Minimum resolution at different frequencies. (**b**) Minimum resolution under different resistivities.

As shown in Figure 3a, when the resistivity values are 10, 100, 1000, and 10,000 Ω·m, the minimum vertical resolution decreases with increasing transmission frequency at fixed frequency intervals, thereby enhancing the imaging resolution. Figure 3b further illustrates that under a constant resistivity condition, increasing the transmission frequency into the megahertz range enables the transmitter to support millimeter-scale high-resolution imaging across a wide range of resistivity values. However, as the frequency increases, the switching losses of the transmitter’s power electronic devices become significantly more pronounced. This underscores the critical importance of appropriate device selection and careful transmitter circuit design.

## 3. Design of the Controlled-Source Electromagnetic Transmitter Circuit

### Power Circuit Design

**Figure 4:**
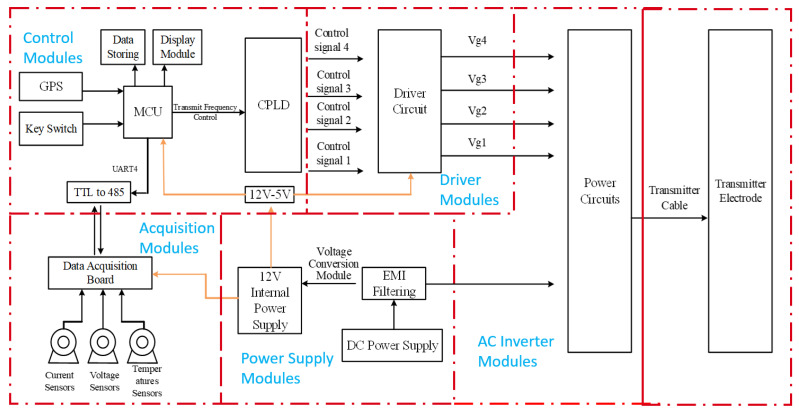
Overall hardware architecture of the high-efficiency miniaturized transmitter.

**Figure 5:**
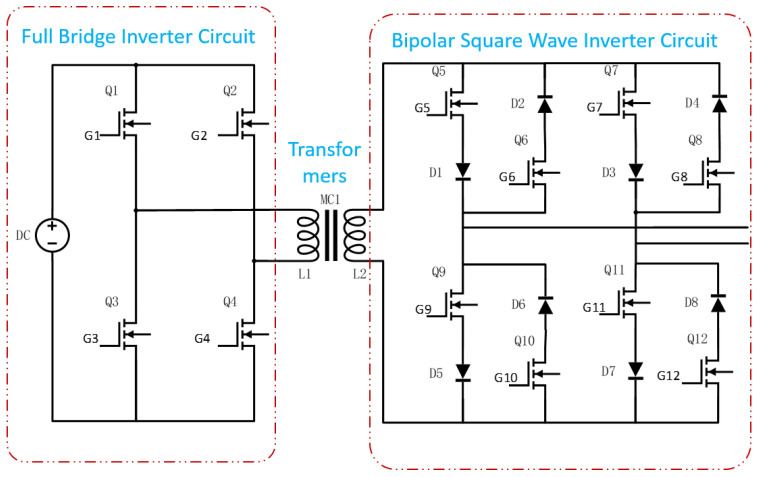
Schematic diagram of the power circuit design for the high-efficiency miniaturized transmitter.

The overall hardware architecture of the transmitter is shown in Figure 4. It primarily consists of a control module, driver module, signal acquisition module, power supply module, and power circuit module. The power circuit, detailed in Figure 5, includes a full-bridge inverter, a high-frequency transformer, and a bipolar square-wave inverter.

SiC-MOSFETs (ADP120N080G2) are employed as the main switching devices, as shown in Figure 5 for Q1 to Q12, and 20A10 is selected as the diode.

The full-bridge inverter circuit serves as the front-end circuit of the transformer’s primary side, generating an AC output voltage with an amplitude equal to the DC bus voltage through precise timing control of the four switches. This configuration enables the circuit to deliver a relatively high output voltage. Meanwhile, using high-voltage-rated switching devices allows the system to handle large currents, enabling a high-power output.

The transformer is constructed using single-strand enameled wire, a low-frequency ferrite core, a BDV0PQ050001 bobbin, and 2UEWF Φ0.1 × 200C wire. This design ensures stable, high-power transmission under high-frequency operation. The key transformer parameters are listed in Table 1.

The bipolar square-wave inverter circuit consists of eight switching devices and eight diodes, which together prevent short-circuits during input voltage polarity reversals. The primary function of this circuit is to directly convert the AC square-wave voltage from the secondary side of the transformer into a higher-frequency output. A Complex Programmable Logic Device (CPLD) is used to generate the drive signals required for frequency conversion. To facilitate voltage-frequency control and ensure high-quality high-frequency square-wave output, the duty cycle of the driving control signal in this design has been intentionally maintained at approximately 50%.

The control signals are divided into two sets for each side of the transformer. On the primary side, Vg1 controls the switching of Q1 and Q4, while Vg2 controls Q2 and Q3. On the secondary side, Vg3 controls Q5, Q6, Q11, and Q12, and Vg4 controls Q7, Q8, Q9, and Q10.

During operation, Q1/Q4 and Q2/Q3 are driven in a complementary manner to invert the DC voltage into an AC square-wave signal. After being stepped up by the transformer, the signal is further processed by complementary switching of Q5/Q6/Q11/Q12 and Q7/Q8/Q9/Q10 to achieve frequency conversion.

**Figure 6:**
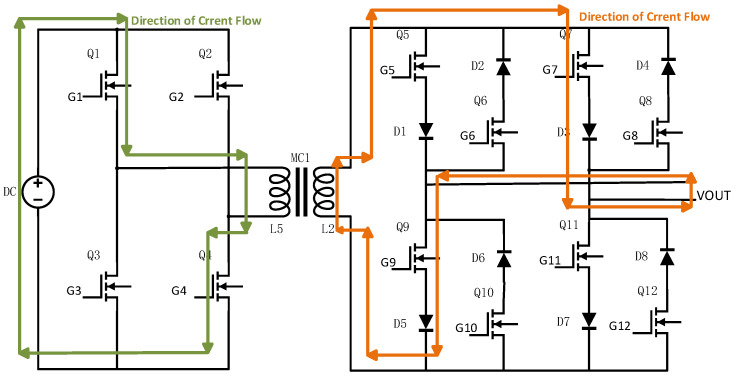
Current flow diagram when VOUT is positive.

**Figure 7:**
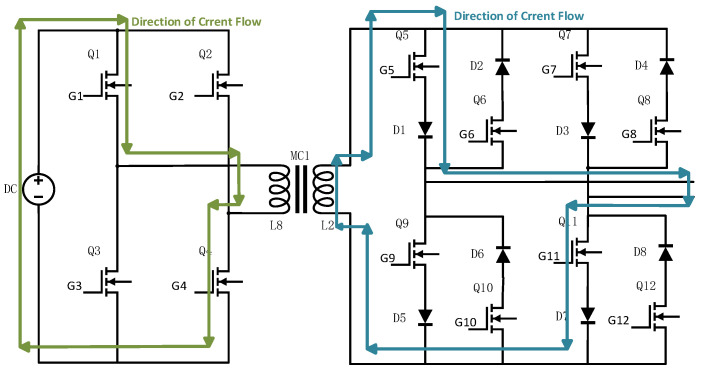
Current flow diagram when VOUT is negative.

For example, when Q1 and Q4 are turned on while Q2 and Q3 are off, the current on the secondary side of the transformer flows upward (defined as the positive direction). If a positive VOUT is desired at this moment, Vg3 is configured to turn off Q5 and Q11, while Vg4 turns on Q7 and Q9, as illustrated in Figure 6. Conversely, to obtain a negative VOUT, Vg3 turns on Q5 and Q11, and Vg4 turns off Q7 and Q9, as shown in Figure 7. Similarly, when Q2 and Q3 are on and Q1 and Q4 are off, the current direction on the secondary side is reversed, and Vg3 and Vg4 are adjusted accordingly to maintain AC square-wave output at VOUT.

By adjusting the duty cycles of the drive control signals Vg1, Vg2, Vg3, and Vg4, the bipolar square-wave inverter circuit can generate output signals of arbitrary frequency.

**Figure 8:**
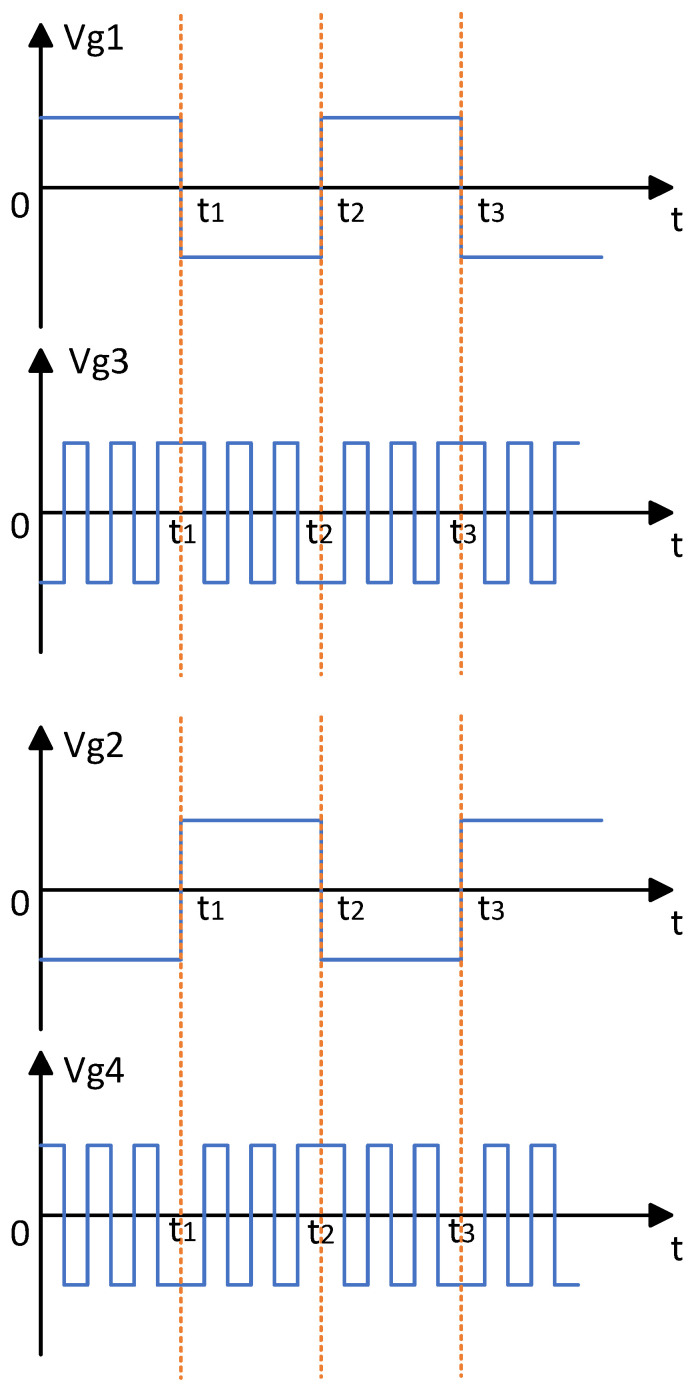
Drive voltage control signals Vg1, Vg2 for full-bridge inverter/Vg3, Vg4 for bipolar square-wave inverter.

To achieve AC frequency conversion, the control signals Vg3 and Vg4 must be synchronized in timing with Vg1 and Vg2. As shown in Figure 8, taking a frequency multiplication factor of six as an example, Vg3 and Vg4 are set to operate at six times the frequency of Vg1 and Vg2, thereby enabling the bipolar square-wave inverter to perform a sixfold frequency conversion of the transformer’s secondary voltage.

**Figure 9:**
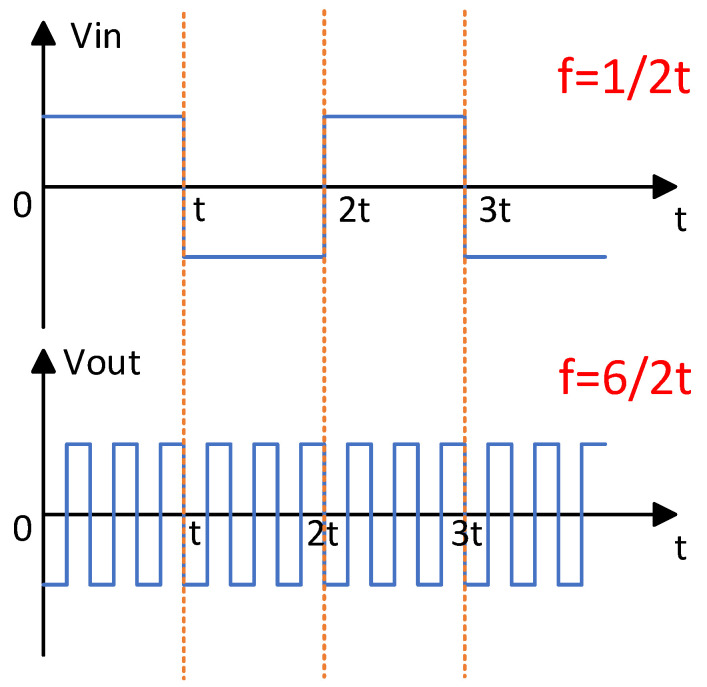
Input/output waveforms of the bipolar square-wave inverter circuit.

The core technique of the circuit design lies in achieving frequency conversion of the AC voltage. During polarity transitions—when conduction switches between Q1/Q4 and Q2/Q3—it is essential that Q5, Q6, Q11, and Q12 and Q7, Q8, Q9, and Q10 maintain their high-frequency switching operation while also preserving their voltage level at the moment of input polarity reversal. In other words, the inverter must retain the pre-transition logic state for one cycle, as illustrated in Figure 9. This ensures stable frequency conversion of the low-frequency AC signal.

## 4. Circuit Efficiency Analysis

### 4.1. Comparative Analysis Between the Bipolar Square-Wave Inverter and Traditional Transmitter Topologies

**Figure 10:**
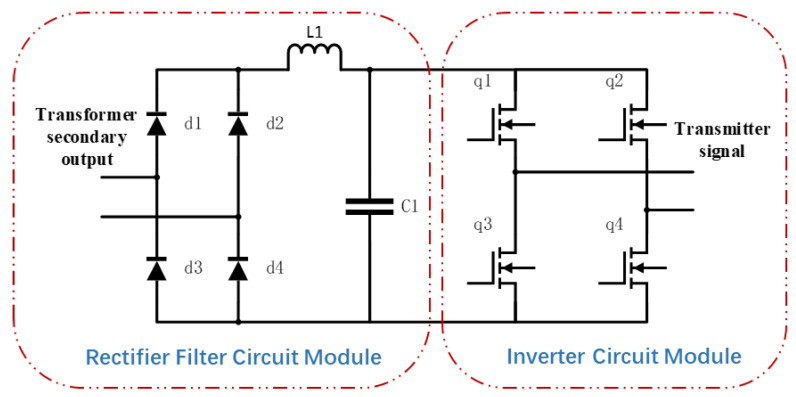
Power circuit diagram of the transformer secondary side in a conventional high-frequency transmitter.

The conventional topology used in high-frequency transmitters on the transformer’s secondary side is illustrated in Figure 10. In this design, the AC voltage from the transformer’s secondary side is first rectified into a pulsating DC signal via a diode bridge. This is followed by smoothing through a large-capacity filter composed of inductor L1 and capacitor C1, generating a DC bus voltage that is subsequently inverted again by a full-bridge inverter to produce the desired AC output at the target frequency. Conventional designs require filtering inductors and capacitors to withstand low-frequency, high-ripple currents, resulting in bulky components and significant copper and core losses in the inductors. Furthermore, the two-stage conversion process introduces additional losses, including rectifier bridge conduction voltage drops and inverter switching losses, limiting overall system efficiency (typically < 95%).

**Figure 11:**
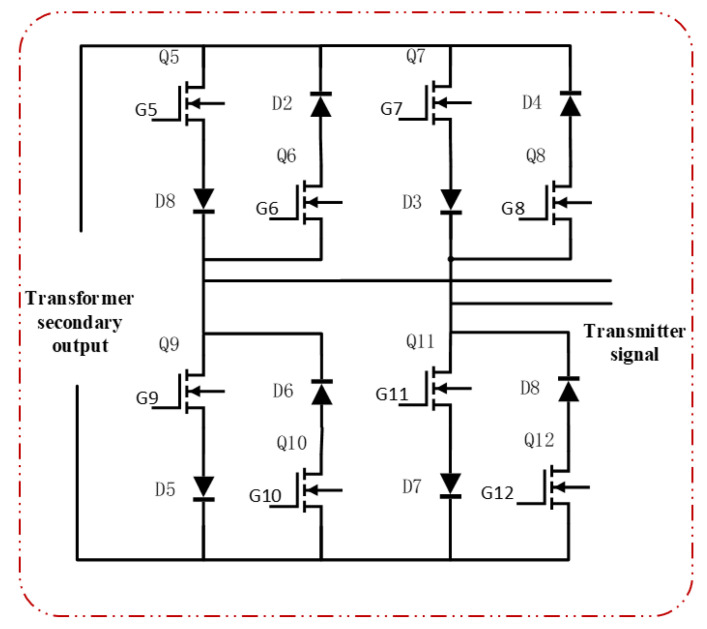
Schematic diagram of the bipolar square-wave inverter circuit.

In contrast, the proposed bipolar square-wave inverter circuit, depicted in Figure 11, enables the direct inversion of the AC voltage from the transformer secondary winding into a higher-frequency signal. This design eliminates the need for large inductors and capacitors. During operation, the eight SiC-MOSFETs and diodes—constituting four bridge legs—alternately conduct, generating the high-frequency AC signal. At identical power levels, the conduction time per switching device is halved compared to conventional approaches. This reduction decreases the conduction losses and junction temperature fluctuation in the switching devices, thereby enhancing circuit efficiency and reliability.

### 4.2. Theoretical Efficiency Analysis of the Bipolar Square-Wave Inverter Circuit

The primary power losses in the bipolar square-wave inverter circuit are attributed to the eight SiC-MOSFETs and associated diodes. At an ambient temperature of T = 25 °C, with a transformer secondary output voltage $V_{DD}$ = 800 V, gate voltage $V_{GS}$ = 20 V, drain current $I_{D}$ = 20 A, and gate resistance $R_{on}$ = 0 Ω, the device datasheet specifies the following characteristics for the SiC-MOSFETs:turn-on energy loss $E_{on}$ = 748.8 μJ, turn-off energy loss $E_{off}\;$= 31.2 μJ, on-state resistance $R_{ds(on)}$ = 80 mΩ, and total gate charge $Q_{g}$ = 58 nC.

As shown in Figure 6 and Figure 7, two SiC-MOSFETs and two diodes conduct simultaneously to produce each output signal cycle at VOUT. At an operating frequency of 10 kHz and a drive control duty cycle of 0.5, the switching energy lost per second for each SiC-MOSFET can be calculated as follows:

(8) $$P_{sw}=\frac{E_{on+off}}{t}=\frac{\left(E_{on}+E_{off}\right)\times 10,000\times 2}{1s}=15.6\;w$$

The conduction loss $P_{on}$ is given by

(9) $$P_{on}={I_{D}}^{2}\times R_{ds\left(on\right)}\times 2=64\;w$$

The gate driving loss $P_{g}$ is given by

(10) $$P_{g}=Q_{g}\times V_{GS}\times f\approx 0.01\;w$$

The reverse recovery loss $P_{rr}$ is given by

(11) $$P_{rr}=V_{DD}\times Q_{rr}\times f\times 2=31.2\;w$$

Therefore, the total power loss of the SiC-MOSFETs in the bipolar square-wave inverter circuit $P_{mosfet}$ is given by

(12) $$P_{mosfet}=P_{sw}+P_{on}+P_{g}+P_{rr}=110.81\;w$$

In this operating state, the diode’s loss is mainly caused by conduction loss, while switching loss and reverse recovery loss can be ignored. According to the chip manual, when $I_{D}$ = 20 A, the maximum forward voltage $V_{F}$ of the diode is 1.1 V. Therefore, the diode loss $P_{diode}$

(13) $$P_{diode}={V_{F}\times I}_{D}\times 2=44\;w$$

Therefore, the total power loss of the bipolar square-wave inverter circuit $P_{total}$ is given by

(14) $$P_{total}=P_{mosfet}+P_{diode}=154.81\;w$$

Under this operating condition ($V_{DD}$ = 800 V, $I_{D}$ = 20 A, D = 0.5), the theoretical operating efficiency η is approximately as follows:

(15) $$\eta =(\frac{V_{DD}\times D\times I_{D}}{V_{DD}\times D\times I_{D}+P_{total}})\times 100\%=98.1\%$$

Therefore, the bipolar square-wave inverter circuit significantly enhances the operating efficiency of the transmitter.

## 5. Simulation and Experimental Validation

### 5.1. Simulation Verification

**Figure 12:**
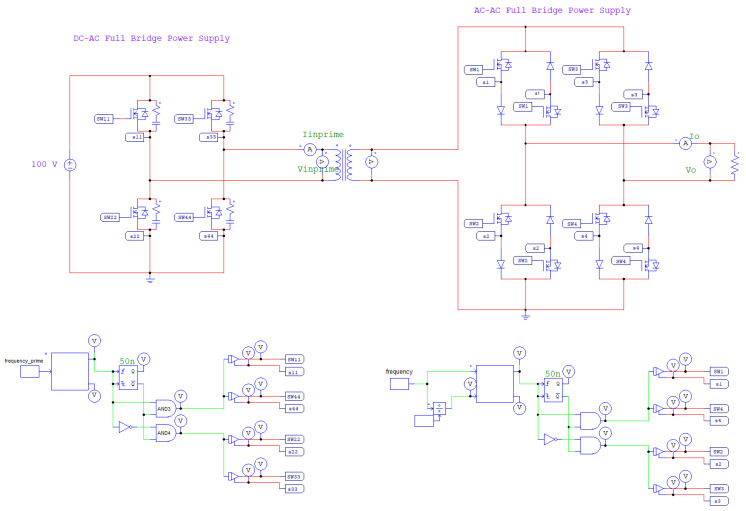
PSIM simulation schematic of the bipolar square-wave inverter circuit.

The performance of the transmitter was verified through simulations conducted in PSIM. The schematic diagram is shown in Figure 12. The turns ratio of the primary to secondary windings of the transformer is set to 1:3, resulting in a threefold voltage amplification at the primary stage.

**Figure 13:**
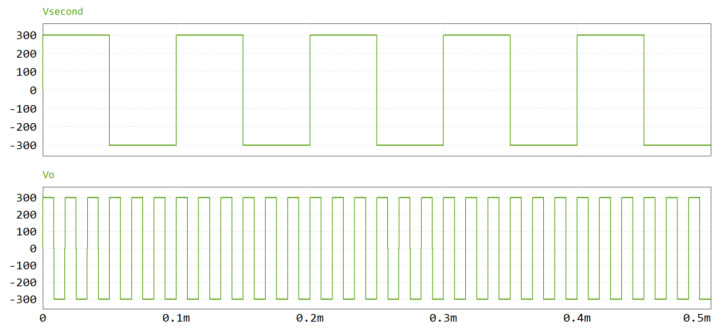
Comparison waveform of front-end voltage and output voltage.

Using a frequency multiplication factor of six as an example, the input voltage is set to 100 V. After inversion and step-up through the front-end circuit and transformer, an AC square-wave output with a peak amplitude of 300 V (V-second) is obtained. The bipolar square-wave inverter then converts V-second to an output voltage V_0_, whose frequency is six times that of the original signal, as shown in Figure 13. These results confirm that the designed circuit successfully achieves frequency conversion of the AC square-wave signal.

### 5.2. Laboratory Transmission Testing

**Figure 14:**
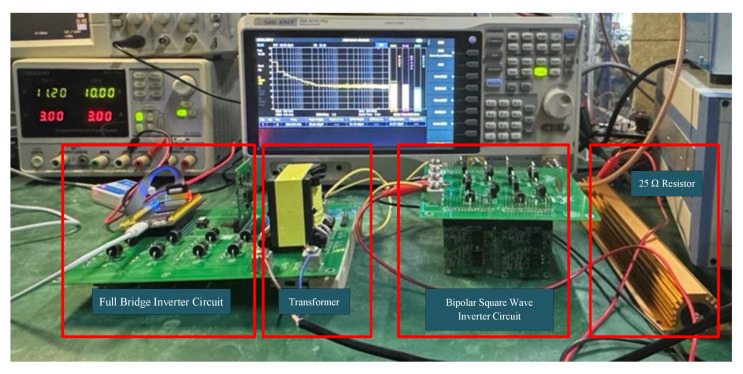
Test setup of the electromagnetic transmitter.

Systematic testing and analysis of the high-frequency electromagnetic transmitter based on the bipolar square-wave inverter circuit were conducted in a laboratory environment. The test setup is illustrated in Figure 14. The experimental system adopts a two-stage inverter topology: the front-end consists of a full-bridge inverter, and the back-end is configured with a bipolar square-wave inverter circuit.

During the tests, the system input voltage was set to 10 V DC, and a 25 Ω equivalent resistance was used at the output to simulate a grounded load.

**Figure 15:**
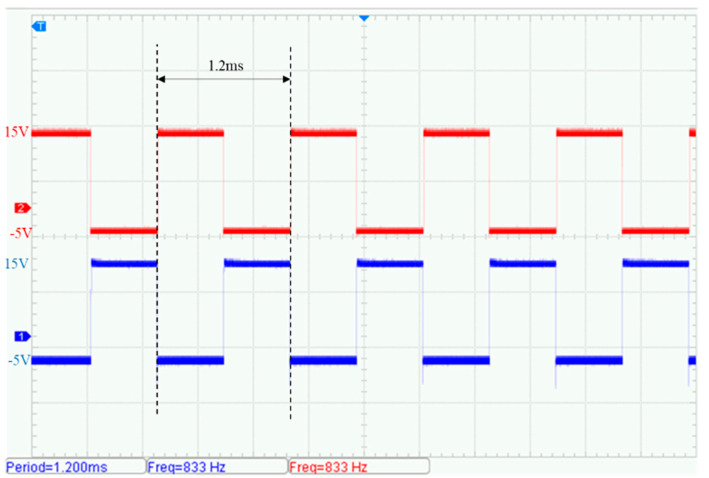
Measured gate drive voltage waveforms of the full-bridge inverter: Vg1 (red), Vg2 (blue).

**Figure 16:**
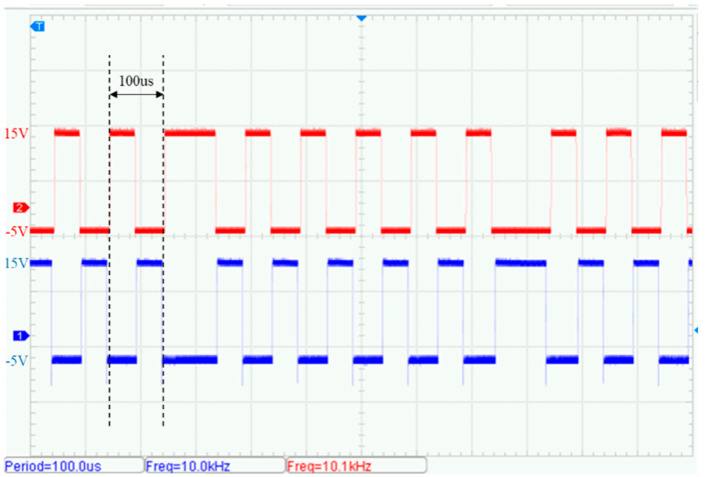
Measured gate drive voltage waveforms of the bipolar square-wave inverter: Vg3 (red), Vg4 (blue).

Taking a 10 kHz transmission signal as an example, the control signal test results are shown in Figure 15 and Figure 16. The measured gate drive signals Vg1 and Vg2 for the front-end inverter circuit exhibit a frequency of 833 Hz, slightly deviating from the theoretical design value of 1 kHz. This frequency offset is primarily attributed to the configured dead time for the power switching devices (typically 400 ns). The signals are bipolar, swinging from +15 V to –5 V, and maintain a duty cycle of 50%.

**Figure 17:**
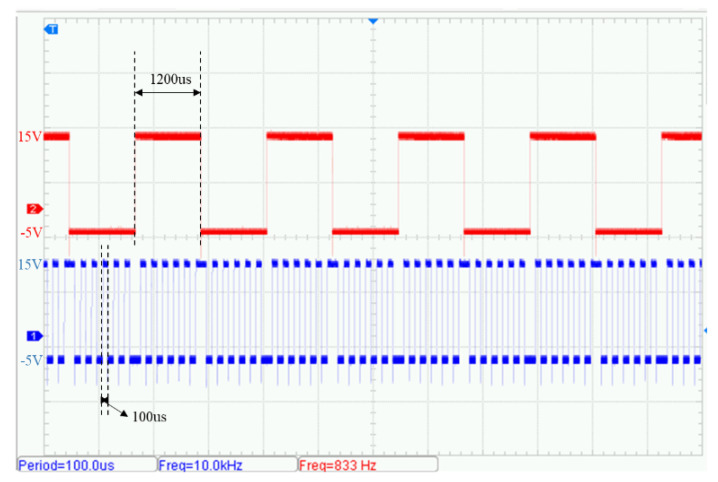
Comparison of measured gate drive signals: Vg1 (red) vs. Vg3 (blue).

The signals Vg3 and Vg4 in the back-end inverter operate at a tenfold frequency relative to the front-end control signals, with a measured frequency of 10.02 kHz ± 0.5%. Their voltage amplitude characteristics match those of the front-end signals. As shown in Figure 17, a strict synchronization mechanism is maintained between the two levels of control signals. While Vg3 operates at a fixed switching frequency of 10 kHz, its transition edges are phase-locked to those of Vg1, resulting in a composite modulated waveform.

**Figure 18:**
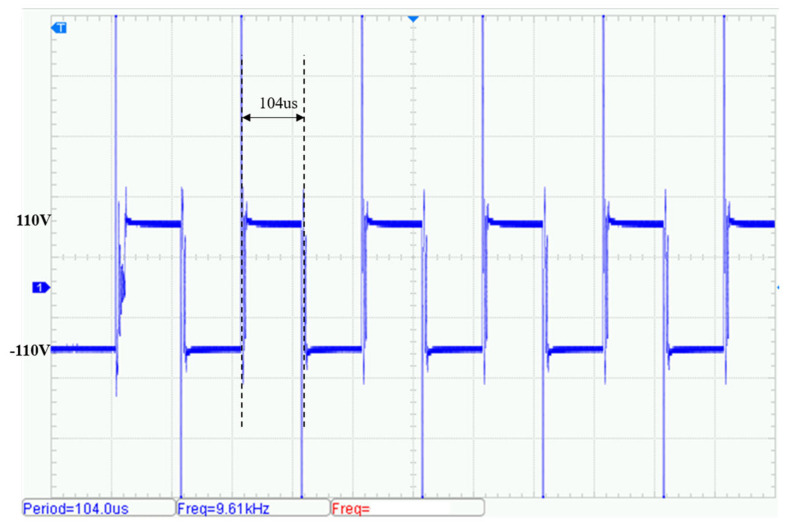
Voltage waveform of transmit signal (10 kHz).

The measured transmission voltage waveform is presented in Figure 18. The output frequency is approximately 9.6 kHz (within ±0.5% of 10 kHz), with a transmission current of 0.87 A. The measured circuit efficiency is approximately 91.1%. Noticeable overshoot appears on both the rising and falling edges of the waveform, with peak transient voltages reaching ±32 V. This is primarily due to resonance between the power-loop parasitic inductance and the SiC-MOSFETs’ junction capacitances. Ongoing optimization efforts are underway to mitigate this effect.

**Figure 19:**
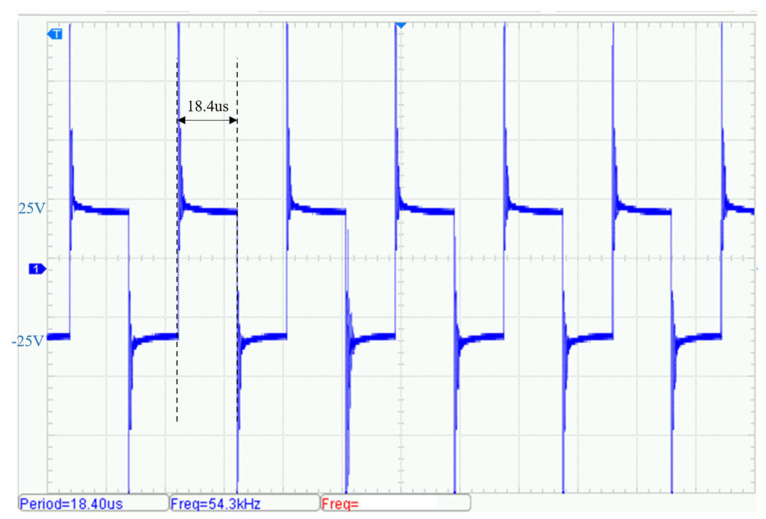
Voltage waveform of transmit signal (50 kHz).

**Figure 20:**
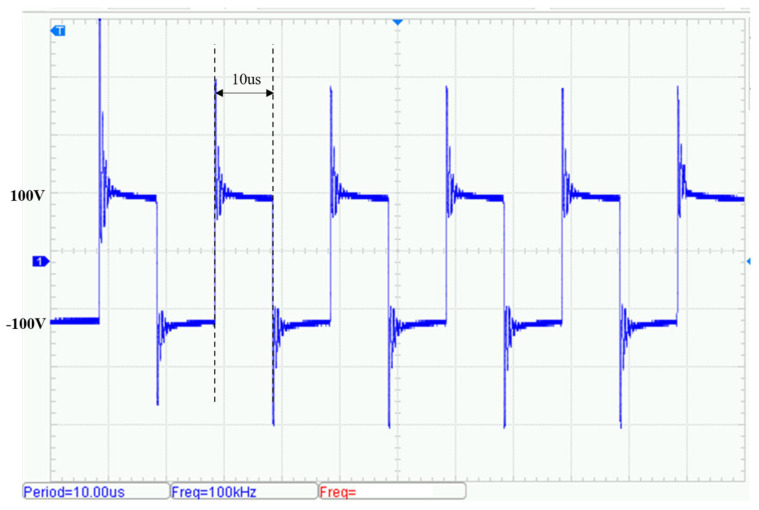
Voltage waveform of transmit signal (100 kHz).

By adjusting the drive control signal frequencies, additional transmission tests were conducted at 50 kHz and 100 kHz. Figure 19 shows the output waveform at 50 kHz. The circuit efficiency measured under the same experimental conditions was approximately 90.2% and Figure 20 shows the output at 100 kHz. The circuit efficiency measured under the same experimental conditions was approximately 89.8%. Although the amplitude of transient voltage spikes increases at higher frequencies, the fundamental component of the waveform remains stable and the total harmonic distortion (THD) stays below 5%.

These laboratory tests with a 25 Ω resistive load demonstrate that the designed high-frequency electromagnetic transmitter exhibits excellent stability across the 10–100 kHz range under indoor (simulated load) conditions.

### 5.3. EMI Near-Field Radiation and Conducted Emission Testing

To evaluate whether the high-frequency transmitter meets international electromagnetic compatibility (EMC) standards, EMI near-field radiation and conducted emission tests were carried out under laboratory conditions. During testing, the transmitter operated at a signal frequency of 100 kHz with an output current of 0.2 A.

**Figure 21:**
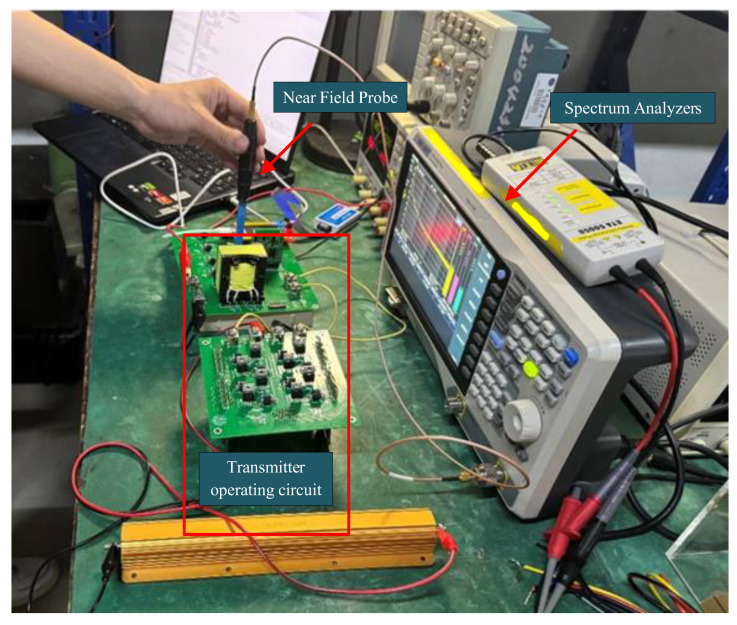
Near-field radiation test setup of the transmitter.

**Figure 22:**
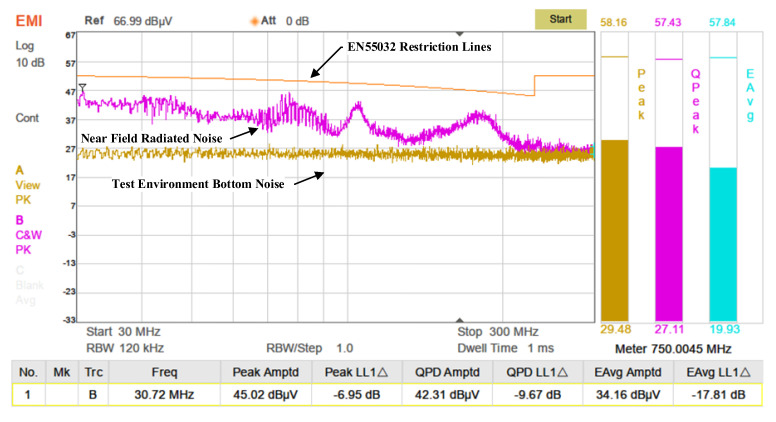
Measured near-field radiation noise spectrum of the transmitter.

For near-field radiation testing, a near-field probe connected to a spectrum analyzer was used to measure electromagnetic interference. The probe captures electric and magnetic field emissions from interference sources and converts them into electrical signals for analysis by the spectrum analyzer. As shown in Figure 21, the analyzer’s sweep frequency range was set from 30 MHz to 300 MHz. While the transmitter was operating, the near-field probe was moved across the circuit, and stronger interference signals were detected near the MOSFETs. Background noise was recorded with the transmitter turned off, and radiated noise was measured again after the system stabilized upon reactivation. The test results are shown in Figure 22. The yellow background line represents the EN55032 Class A industrial equipment near-field radiation limit. The environmental noise floor was approximately 25 dBμV, with more prominent radiation noise observed at the lower end of the frequency spectrum.

**Figure 23:**
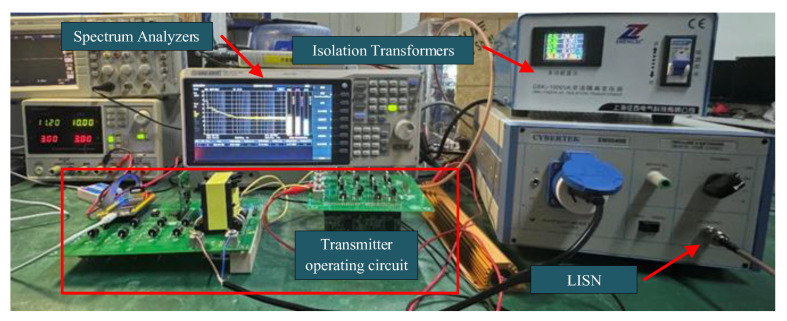
Conducted emission test setup of the transmitter.

**Figure 24:**
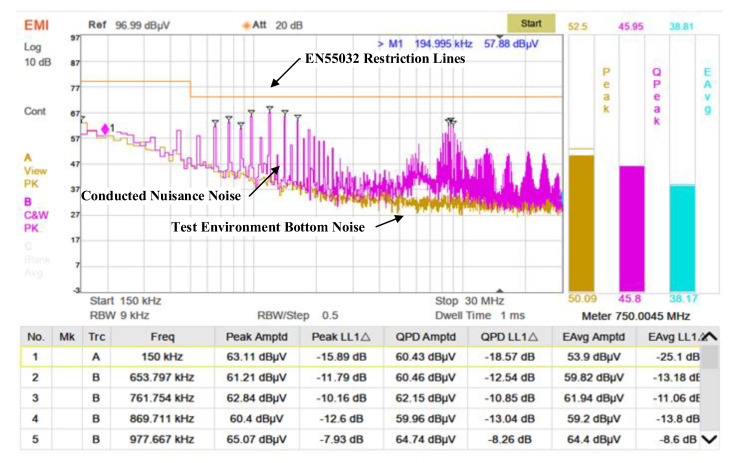
Measured conducted emission noise spectrum of the transmitter.

For conducted emission testing, a setup comprising a spectrum analyzer, LISN (Line Impedance Stabilization Network), and an isolation transformer was used, as illustrated in Figure 23. The analyzer’s sweep range was configured from 150 kHz to 30 MHz. Background noise from the power line, LISN, and surrounding environment was measured with the transmitter turned off. The transmitter was then activated, and the LISN coupled the interference signals generated by the transmitter through the power and signal lines to the spectrum analyzer. The measured conducted emission results are shown in Figure 24. The yellow background line indicates the EN55032 Class A conducted emission limit. All quasi-peak values of conducted noise were below the specified limits.

These results confirm that the developed high-frequency transmitter complies with the EN55032 Class A EMI standard for industrial equipment, demonstrating compliance with international electromagnetic compatibility requirements and the capability for safe, stable operation in real-world environments.

## 6. Joint Field Survey with High-Frequency Receiver

**Figure 25:**
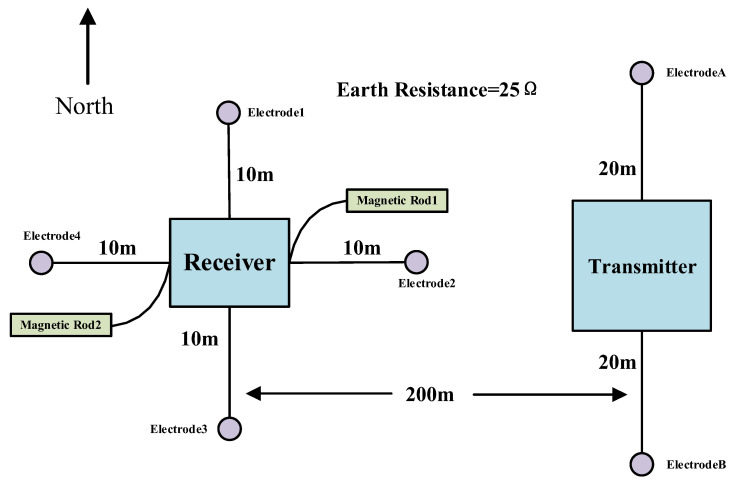
Field test schematic diagram.

To verify the actual performance of the developed transmitter, we conducted field exploration tests in Tongzhou District, Beijing, using the electromagnetic transmitter we developed and the high-frequency receiver developed by our research group. Measurements indicated that the ground resistance at the test site was approximately 25 Ω. Aluminum foil was used as the transmitter electrode and connected to the transmitter via cables. As shown in Figure 25, in this experiment, the transmitter was positioned 20 m away from the electrode, with a distance of 40 m between transmitter electrodes A and B.

**Figure 26:**
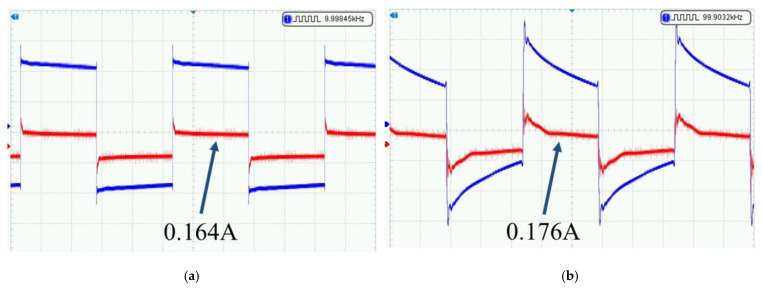
Measured waveforms of transmitted signals. (**a**) Minimum resolution at different frequencies; (**b**) minimum resolution under different resistivities.

During the experiment, signals in the 10–100 kHz frequency range were transmitted. The receiver electrodes were arranged 10 m away from the transmitter in the four cardinal directions (north, south, east, and west), with two magnetic rods placed around them. The distance between the transmitter and receiver was 200 m. Taking signals at 10 kHz and 100 kHz as examples, the measured transmission signals during the experiment are shown in Figure 26. When transmitting a 10 kHz signal, the peak transmission current was approximately 0.164 A, and when transmitting a 100 kHz signal, the peak transmission current was approximately 0.176 A.

**Figure 27:**
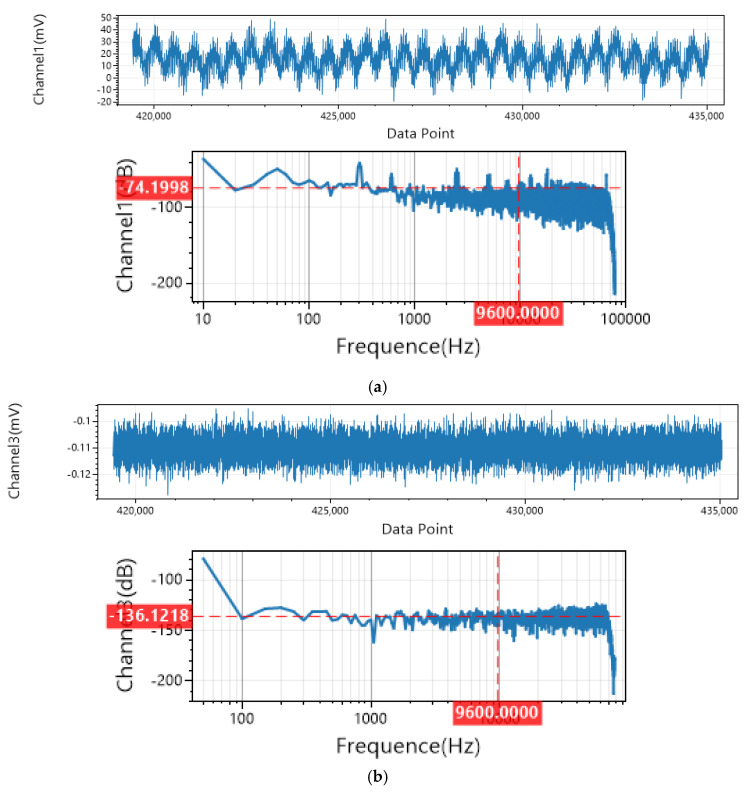

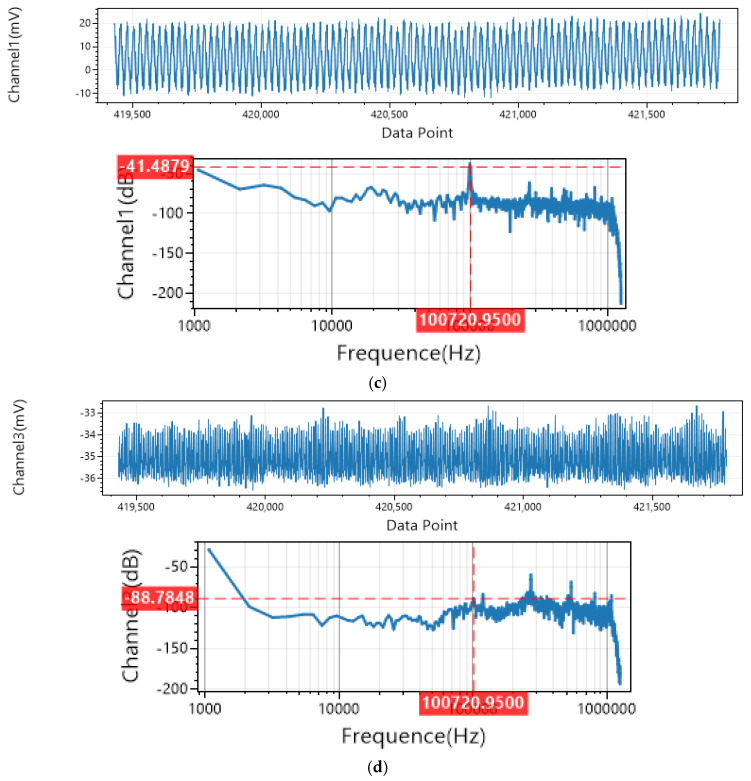
Time–frequency spectra of signals received by the high-frequency receiver. (**a**) Time–frequency spectrum of 10 kHz signal received by high-frequency receiver (north–south direction). (**b**) Time–frequency spectrum of 10 kHz signal received by high-frequency receiver (east–west direction). (**c**) Time–frequency spectrum of 10 kHz signal received by high-frequency receiver (east–west direction). (**d**) Time–frequency spectrum of 100 kHz signal received by high-frequency receiver (east–west direction).

Analysis of the measured data from the high-frequency receiver shows a significant correlation between the time–frequency response characteristics of the north–south (NS) and east–west (EW) electric field signals. As illustrated in Figure 27, stable pulsed signal features appear in the time–frequency spectrograms at the system’s operating frequencies (10 kHz and 100 kHz). The time-domain waveforms and spectral characteristics of the dual-channel electric field signals exhibit a high degree of consistency, with a correlation coefficient of 0.92. The field tests confirmed that the developed transmitting system successfully covers the 10–100 kHz range, delivering an output signal with a signal-to-noise ratio (SNR) of 45 dB. These performance metrics satisfy the technical requirements for frequency-domain electromagnetic exploration equipment in complex geological environments.

## 7. Conclusions

This study presents a compact high-frequency electromagnetic transmitter based on a bipolar square-wave inverter topology.

(1) For the first time, a novel bipolar square-wave inverter topology is proposed. This architecture replaces the traditional DC–DC boost stage and multi-stage inversion by directly converting the transformer’s secondary-side AC square-wave output. By eliminating the rectification and filtering stages, the circuit is significantly simplified, laying the foundation for miniaturization and high efficiency.
(2) Laboratory tests with a 25 Ω resistive load, as well as field surveys with a high-frequency receiver, confirm that the transmitter can reliably output square-wave signals across the 10–100 kHz range, achieving an efficiency of up to 92%.
(3) EMI near-field radiation and conducted emission tests demonstrate that the transmitter complies with the EN55032 Class A standard for industrial equipment.

Building on the current design, future work will focus on further miniaturization—aiming to reduce overall volume and weight by an additional 20%—and on increasing the stable operating frequency to 200 kHz. These advancements will further improve system efficiency and provide a lighter, highly reliable solution for urban subsurface imaging with millimeter-scale resolution.

## Figures and Tables

**Table 1:** Design parameters of the high-frequency transformer.

Parameter	Core (Bobbin) Model	Operating Frequency	Maximum Input Voltage	Maximum Output Voltage/ Current	Reverse Breakdown Voltage of Switch	Maximum Duty Cycle	Minimum Duty Cycle	Voltage Withstand Rating	Turns Ratio
Design Specification	BDV0PQ050001	100 kHz	50 V	150 V/15 A	600 V	0.8	0.2	600 V	1:3

## Data Availability

The raw data supporting the conclusions of this article will be made available by the author, Zhongping Wu, on request.

## References

[1] Ye W., Huang J., Xu P., Yuan J., Zeng L., Zhang Y., Wang Y., Wang S., Xu X., Guo Z. (2025). Suitability Evaluation of Underground Space Development by Considering Socio-Economic Factors—An Empirical Study from Longgang Region of China. Sustainability.

[2] Yang M., Zhu Y., Ji X., Wang J., Fang H. (2025). Study on Development Pattern and Comprehensive Evaluation of Integration of Urban Underground Space and Rail Transit in China. Sustainability.

[3] Zhou X., Liu S., Chen A., Chen Q., Xiong F., Wang Y., Chen H. (2023). Underground Anomaly Detection in GPR Data by Learning in the C3 Model Space. IEEE Trans. Geosci. Remote Sens..

[4] Zonge K.L., Hughes L.J., Nabighian M.N. (1991). Controlled source audio-frequency magnetotellurics (CSAMT). Electromagnetic Methods in Applied Geophysics: Volume 2, Application, Parts A and B.

[5] Cheng S., Zhang Z.-Y., Zhou F., Li M., Chen H., Shi F.-S., Huang L.-P., Li Y. (2021). 3D Step-by-step inversion strategy for audio magnetotellurics data based on unstructured mesh. Appl. Geophys..

[6] Xu Z., Liao X., Liu L., Fu N., Fu Z. (2023). Research on Small-Loop Transient Electromagnetic Method Forward and Nonlinear Optimization Inversion Method. IEEE Trans. Geosci. Remote Sens..

[7] Sanny T.A. (2017). Identification of Lembang fault, West-Java Indonesia by using controlled source audio-magnetotelluric (CSAMT). AIP Conf. Proc..

[8] Zhang J., Zeng Z., Zhao X., Li J., Zhou Y., Gong M. (2020). Deep mineral exploration of the jinchuan Cu–Ni sulfide deposit based on aeromagnetic, gravity, and csamt methods. Minerals.

[9] Hu X., Peng R., Wu G., Wang W., Huo G., Han B. (2013). Mineral Exploration using CSAMT data: Application to Longmen region metallogenic belt, Guangdong Province, China. Geophysics.

[10] Zhang K., Lin N., Wan X., Yang J., Wang X., Tian G. (2022). An approach for predicting geothermal reservoirs distribution using wavelet transform and self-organizing neural network: A case study of radon and CSAMT data from Northern Jinan, China. Geomech. Geophys. Geo-Energy Geo-Resour..

[11] Tao H., Yang N., Wang H. (2024). Cascaded controllable source circuit and control of electromagnetic transmitters for deep sea exploration. J. Power Electron..

[12] Zhang K., Zhang R., Wang M., Lin Z., Zhang Q., Jing J., Li F., Yang S. (2025). Controlled source ultra-audio frequency magnetotellurics transmitter for high-resolution detection of urban shallow underground space. Measurement.

[13] Winter H. (2023). Mitsubishi Electric to Ship Samples of SBD-embedded SiCMOSFET Module. Electron. Newsweekly.

[14] Walter M., Bakran M.-M. (2025). Enhancing Hybrid Inverter Performance Through Inductive Decoupling of Silicon and Silicon Carbide Devices. IEEE Trans. Ind. Appl..

[15] Zhu P., Liu X., Yu Z., Zhao L., Li X. (2023). Application of phase-shifted full-bridge soft-switch technology in suspension chopper. Meas. Control..

[16] Cheng X., Liu C., Wang D., Zhang Y. (2021). State-of-The-Art Review on Soft-Switching Technologies for Non-Isolated DC-DC Converters. J. IEEE Access..

[17] Yang F., Lv Z., Song Z., Li H., Zhang Z., Dong B. (2025). Optimization Design of Magnetic Integrated Planar Transformer for Bidirectional CLLC Resonant Converter. J. Electr. Eng. Technol..

[18] Liu C., Qi L., Cui X. (2025). Design Considerations on Voltage and Current Transfer Ratio of High-Frequency Transformers. CSEE J. Power Energy Syst..

[19] Xie M., Tan H., Wang K., Guo C., Zhang Z., Li Z. (2017). Study on the characteristics of 3D CSAMT tensor impedance data. Prog. Geophys..

[20] Zhen Q.-H., Di Q.-Y., Liu H.-B. (2013). Key technology study on CSAMT transmitter with excitation control. Chin. J. Geophys.-Chin. Ed..

[21] Liu X., Gao S. (2024). Response characteristics of 3D tensor CSAMT in axis anisotropic media. Front. Earth Sci..

[22] Hasan M., Su L., Cui P., Shang Y. (2025). Development of deep-underground engineering structures via 2D and 3D RQD prediction using non-invasive CSAMT. Sci. Rep..

